# Cyclic dinucleotides mediate bacterial immunity by dinucleotide cyclase in *Vibrio*

**DOI:** 10.3389/fmicb.2022.1065945

**Published:** 2022-12-22

**Authors:** Zengzeng Lu, Yuqian Fu, Xueyuan Zhou, Hekang Du, Qi Chen

**Affiliations:** Fujian Key Laboratory of Innate Immune Biology, Biomedical Research Center of South China, Fujian Normal University Qishan Campus, Fuzhou, China

**Keywords:** DncV, cGAS, CDNs, OAS, bacterial immunity

## Abstract

The cyclic GMP-AMP (cGAMP) synthase (cGAS) recognizes cytosolic DNA and synthesizes the second messenger, cGAMP, thus activating the adaptor protein stimulator of interferon genes (STING) and initiating the innate immune responses against microbial infections. cGAS-STING pathway has been crucially implicated in autoimmune diseases, cellular senescence, and cancer immunotherapy, while the cGAS-like receptors in bacteria can protect it against viral infections. Dinucleotide cyclase in *Vibrio* (DncV) is a dinucleotide cyclase originally identified in *Vibrio cholerae*. The synthesis of cyclic nucleotides by DncV, including c-di-GMP, c-di-AMP, and cGAMP mediates bacterial colonization, cell membrane formation, and virulence. DncV is a structural and functional homolog of the mammalian cytoplasmic DNA sensor, cGAS, implicating cGAS-STING signaling cascades may have originated in the bacterial immune system. Herein, we summarize the roles of DncV in bacterial immunity, which are expected to provide insights into the evolution of cGAS-STING signaling.

## 1. Introduction

**Figure 1:**
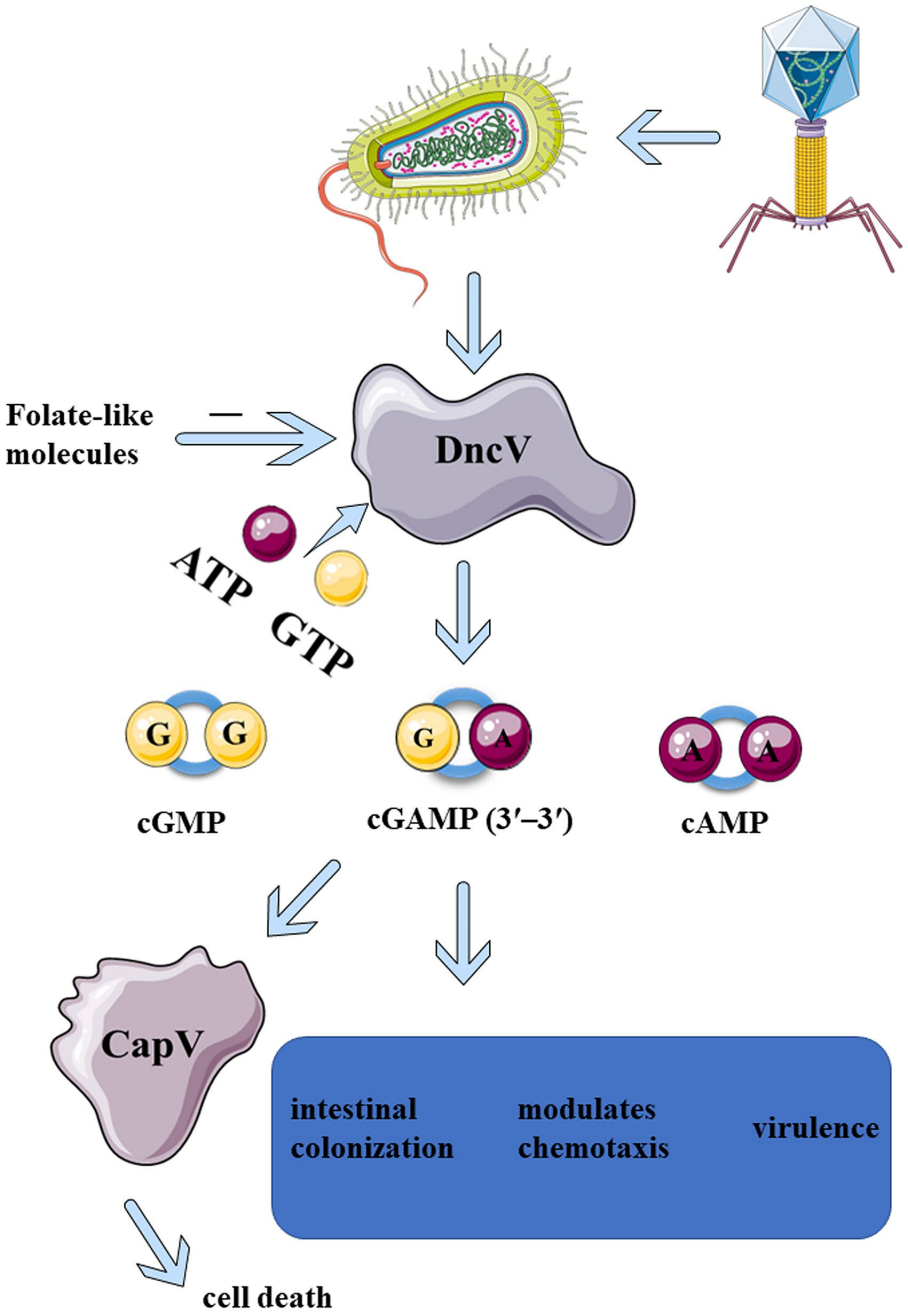
Upon infection of phages, DncV is induced and activated in *Bacteria*, which in turn recruits ATP and GTP to produce CDNs, mainly 3′3′-cGAMP. 3′3′-cGAMP can activate CapV phospholipase, leading to bacterial cell membrane degradation and bacterial death before phage replication is complete. 3′3′-cGAMP is essential for regulating the chemotaxis, virulence, and colonization of *Vibrio cholerae* and other bacteria (Davies et al., 2012). The enzymatic activity of DncV is inhibited by folate-like molecules, which might be the negative feedback mechanism that folate-like metabolism cofactors modulate the synthesis of cyclic dinucleotide second messenger.

“Dinucleotide cyclase in *Vibrio*” (DncV, VC0179) is a dinucleotide cyclase found in bacteria. It catalyzes the generation of cyclic dinucleotides. DncV was first discovered in early 2012 in the toxin-co-regulated pilus (TCP) island, a chromosomal segment encoding several virulence-associated genes found in all the pandemic strains of *Vibrio cholerae*. The DncV enzyme preferentially synthesizes a cyclic dinucleotide 3′3′-cyclic GMP-AMP (cGAMP), c-di-GMP (CDG, cyclic di-GMP), and c-di-AMP (CDA, cyclic di-AMP; Figure 1; Davies et al., 2012). Kellenberger and colleagues have developed RNA-based fluorescent biosensors to detect the *in vivo* production and biological activity of cyclic AMP-GMP (Kellenberger et al., 2013). c-AMP-GMP is synthesized in the presence of all four nucleotide triphosphate esters (Kellenberger et al., 2013). By the end of 2012, 2′3′-cGAMP was identified in mammalian cells and was synthesized by cyclic GMP-AMP synthase (cGAS). cGAS binds to the DNA to generate 2′3′-cGAMP and serves as a cytosolic immune sensor of pathogen DNA. As a second messenger, 2′3′-cGAMP binds to stimulator of interferon genes (STING), thus triggering downstream immune responses (Sun et al., 2013; Wu et al., 2013). The study of DncV is expected to reveal the evolution of cGAS-STING innate immunity, thus linking microbes to the immunity of metazoans.

## 2. Cyclic dinucleotides and DncV

In 1987, c-di-GMP was first identified as an allosteric regulator of cellulose biosynthesis and was the first cyclic dinucleotide to be discovered in bacteria (Ross et al., 1987; Petchiappan et al., 2020). c-di-GMP is a second messenger involved in regulating several physiological functions. It protects *Vibrio cholerae* (*V. cholerae*), *Mycobacterium tuberculosis*, and other bacterial pathogens from severe environmental conditions by regulating cell cycle, cell differentiation, biofilm formation, virogenicity, bacterial colonization, and immune responses (Angeloni et al., 2020; Floyd et al., 2020; Gallagher et al., 2020; Nicastro et al., 2020; Petchiappan et al., 2020; Xu et al., 2020). c-di-GMP is a STING agonist and has been applied as an effective vaccine adjuvant in the combinatorial treatment for melanoma and liver cancer with irreversible electroporation (IRF; Shin et al., 2020; Lasarte-Cia et al., 2021). c-di-GMP triggers strong humoral and cellular immune responses and has a promising prospect and potential as a vaccine adjuvant, especially as a mucosal vaccine adjuvant, and in the combination treatment for cancer.

In 2008, c-di-AMP was serendipitously discovered due to its binding to *Bacillus subtilis* protein, DNA integrity scanning protein A (DisA; Witte et al., 2008). Like c-di-GMP, c-di-AMP is a second messenger distributed widely across bacteria and archaea, which involved in regulating the physiological activities of several bacterial species. Interestingly, c-di-AMP is essential for bacterial growth. Insufficient levels of c-di-AMP lead to bacterial lysis, while excessive amounts are toxic. Its levels play an important role in the formation of biofilms, monitoring DNA damage, and eukaryotic immune responses. c-di-AMP therefore has been used as a vaccine adjuvant to improve vaccine efficiency (Gundlach et al., 2015; Yin et al., 2020). c-di-AMP binds to several receptors, including STING, DEAD-box helicase 41 (DDX41), reductase controlling NF-ĸB (RECON or aldo-keto reductase family 1 member C13, encoded by *Akrlc13*), and ERAdP (CTD nuclear envelope phosphatase 1 regulatory subunit 1 or C16orf69, encoded by *Cneplrl*, formerly *Tmem188*), to activate immune responses (He et al., 2020). RECON binds to c-di-AMP and 3′3′-cGAMP but not to c-di-GMP or 2′3′-cGAMP (McFarland et al., 2017). Since many bacteria utilize c-di-AMP as a second messenger, it is necessary to further confirm its regulatory effects *in vivo* when used as a vaccine or immunomodulator. With an in-depth study of c-di-AMP in host innate and adaptive immunity, the development of c-di-AMP as a target of bacterial vaccine or drug has become a research hotspot.

In early 2012, a novel heterocyclic dinucleotide 3′3′-cGAMP was synthesized using DncVs in *Vibrio cholerae* (Davies et al., 2012; Kellenberger et al., 2013). 2′3′-cGAMP synthesized by the cytosolic nucleic acid sensor, cGAS, was discovered in the metazoans (Sun et al., 2013; Wu et al., 2013). Several studies on 2′3′-cGAMP in metazoans exist, and the cGAS-STING pathway has been critically implicated in inflammatory injury, aging, autoimmune diseases, and cancer immunotherapy (Li and Chen, 2018; Basit et al., 2020; Kwon and Bakhoum, 2020). The production of 3′3′-cGAMP in *Vibrio cholerae* can regulate its virulence along with bacterial chemotaxis. 3′3′-cGAMP is related to exogenous electricity in some bacteria (Kellenberger et al., 2015; Nelson et al., 2015). With the increase of DncV expression, the preferentially synthesized 3′3′-cGAMP severely inhibited the chemotactic ability of *Vibrio cholerae*, thus significantly enhancing the intestinal colonization ability of *Vibrio cholerae* (Figure 1). ToxT was the main virulence factor which directly regulate the expression of DncV. ToxT inhibits the expression of transcription factors encoded by *Vibrio* 7th pandemic island-1 (VSP-1), which in turn controls the expression of several VSP-1 genes, and VSP-1 encodes DncV. DncV induced the catabolism of fatty acids and inhibited the synthesis of fatty acids. Fatty acids have been reported to regulate the activity of ToxT. Although it was found that DncV interacted with ToxT, whether DncV induces fatty acid metabolism to regulate the activity of ToxT remains unclear. It was apparent that DncV overexpression inhibits the growth of *Vibrio cholerae* (Davies et al., 2012). One possibility is that the co-expression of cGAMP-activated phospholipase in *Vibrio* (CapV, “cGAMP-activated phospholipase in *Vibrio*”) and the DncV in *Vibrio cholerae* and *Escherichia coli* sensitized these species to overproduction of cGAMP, resulting in growth inhibition. Basically, 3′3′-cGAMP activates CapV phospholipase activity to target the cell membrane, resulting in the degradation of specific phospholipid components in the cell membrane. CapV and 3′3′-cGAMP jointly regulate and reshape the cell membrane of *Vibrio cholerae* for adaptation to different membrane stresses and modify the bacterial membrane (Severin et al., 2018). Overexpression of the DncV of *E. coli, ECOR31,* increases the concentration of 3′3′-cGAMP, which could regulate cell membrane formation as well as flagella-mediated motility at a certain temperature (Li et al., 2019). Like c-di-GMP, c-di-AMP, and 2′3′-cGAMP, 3′3′-cGAMP not only binds to STING but also to other epigenetic receptors, resulting in a wide range of physiological responses (Table 1; McFarland et al., 2017; Li et al., 2019). All of the above CDNs share a common signaling pathway, which can bind to and activate the central connector, STING, in the cytoplasmic DNA sensing pathway, thereby promoting innate immune responses in mammalian cells by inducing the expression of type I interferon (IFN-I; Woodward et al., 2010; Burdette et al., 2011; Gao et al., 2013). Although several pathways associated with 3′3′-cGAMP have been described above, the activator of 3′3′-cGAMP signaling remains elusive. In 2020, three different regulatory mechanisms mediating the 3′3′-cGAMP signaling pathway were identified (Wright et al., 2020). The first small molecule activator of cyclic adenosine phosphate (cAMP) was discovered for the synthesis of cGAMP. Moreover, a specific phosphodiesterase, HD-GYP, was found in bacteria, which promoted cGAMP degradation. Although the mechanisms of 3′3′-cGAMP degradation remain to be fully comprehended, it has been reported that three phosphodiesterases (PDEs; termed as V-cGAP1/2/3) in *Vibrio cholerae* specifically degrade 3′3′-cGAMP but have no effect on other types of cGAMP (Gao et al., 2015). 3′3′-cGAMP can be linearized by all three V-cGAPs to produce 5′-pApG, which is further hydrolyzed into 5′-ApG by V-cGAP1. V-cGAP1 (VCA0681) was previously reported to be able to hydrolyze c-di-GMP, but not c-di-AMP. It was unclear, though, whether V-cGAP1 can hydrolyze c-di-GMP and 3′3′-cGAMP simultaneously *in vivo*. A recent study confirmed that DncV played a role in mediating the antiviral defense (Wright et al., 2020). In a study on the homolog of DncV, DncV was found to be substituted by an unknown gene (WP-001593458, cdnE, the gene cGAS/DncV-like nucleotidyltransferase in *E. coli*) to synthesize a cyclic UMP-AMP (c-UMP-AMP), a hybrid purine-pyrimidine CDN. cGAS/DncV-like nucleotide transferase (CdnE) is named as such since it may share common ancestry with cGAS and DncV. The reactive enzyme is cGAS/DncV-like nucleotidyltransferase (CD-NTase), which synthesizes special oligonucleotide signals to amplify pathway activation and control downstream effects (Whiteley et al., 2019). Bacteria have evolved defense weapons associated with DncV and its homologs-the cyclic oligonucleotide-based signaling system (CBASS). Bacteriophage-infected bacteria initiate individual suicide mechanisms *via* the CBASS system to prevent the proliferation of bacteriophages, thus facilitating the development of a molecular mechanism of immune defense against bacteriophages in the entire bacterial population (Hobbs et al., 2022). CBASS operons contain a CD-NTase that senses phage replication and catalyzes the synthesis of nucleotide second messenger signals to initiate antiviral defense mechanisms (Athukoralage and White, 2022; Duncan-Lowey and Kranzusch, 2022). All essential components of the mammalian cGAS-STING signaling pathway are functionally shared across the bacterial CBASS (Lowey et al., 2020). All these indicate that DncV in bacteria and metazoans, are closely related and plays an important role in bacterial survival and signaling transduction.

**Table 1:** Comparison between cGAS and DncV.

	DncV	cGAS
Source	Bacteria	Mammal
Structure	Highly similar	
Activation route	Self-activation after phage infection	dsDNA inducer activation
Substrates	ATP, GTP	
Products	3′3′-cGAMP (main product), c-di-AMP, c-di-GMP (produced *in vitro* may not be produced *in vivo*)	2′,3′-cGAMP
Receptors	CapV, RECON, and STING	STING
Functions	Defense against phage infection	Defense against viral and bacterial infections, anti-tumor immunity, inflammation, and autoimmune reactions

## 3. Structural basis of DncV

**Figure 2:**
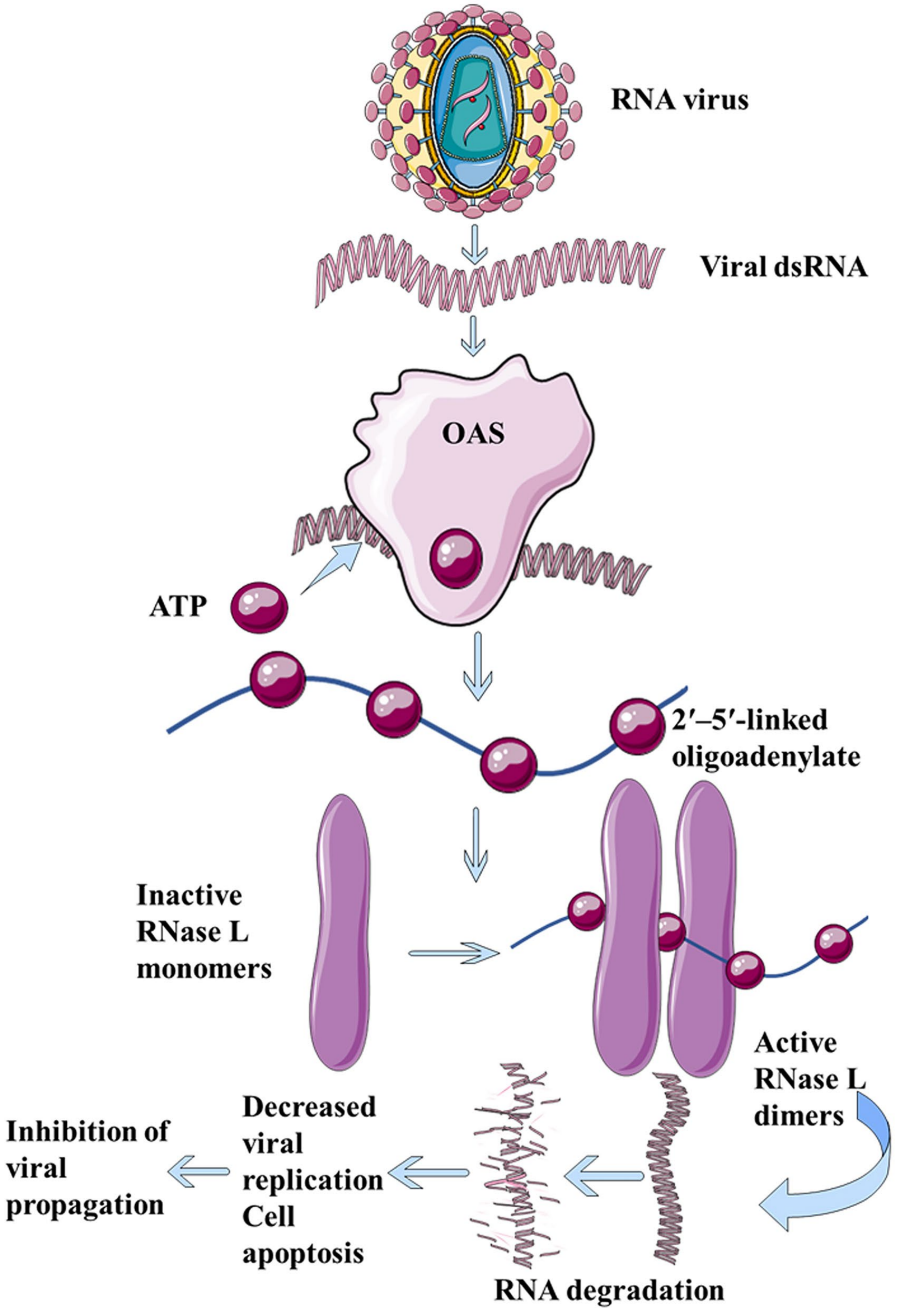
The dsRNA produced during virus replication binds to OAS and catalyzes ATP to form 2′, 5′-linked oligoadenylate, which in turn binds to ribonuclease L, thus forming a cross dimer that degrades cell- and virus-derived RNAs, leading to limitation of viral replication, reduction in cell apoptosis, and inhibition of viral proliferation.

Dinucleotide cyclase in *Vibrio*, human 2′-5′-oligoadenylate synthetases (OAS), and cGAS belong to the large family of nucleotide transferases. OAS is a natural immune sensor of cytoplasmic double-stranded RNA (dsRNA; Schwartz et al., 2020; Figure 2). It is an important antiviral protein that functions to limit viral infection and block the synthesis of toxic proteins. Although DncV and mammalian OAS or cGAS family enzymes share less than 10% sequence identity, DncV is a structural and functional homolog of cGAS and OAS (Kranzusch et al., 2014). Both DncV and cGAS can produce cGAMP, and thus, in this section, we emphasize the similarities and differences between their structures.

The full length of DncV comprises 436 residues and adopts an extended double-lobed structure characterized by nucleotide transferase folding. The bilobed structure comprises a catalytic domain (CD) with an N-terminal α/β core and a C-terminal helical domain (HD). The N-terminal extension and helix α7 of the helical domain combine the helical and catalytic domains. The core of α/β consists of five helices on one side and central twisted β lamellae. The C-terminal helical domain consists of eight helices and a pair of antiparallel β lamellae. There is a deep catalytic pocket between the catalytic and helical structures, while the catalytic ring (residues 112–129) is located at the bottom of the pocket. As a catalytic triad of the nucleotide transferase family, Asp131, Asp133, and Asp193 are the key catalytic aspartic acid residues (Asp193 is spatially proximal to Asp131 and Asp133) located in the β lamellar structure in the α/β core, with two magnesium ions and two nucleotides binding to the active site (Ming et al., 2014; Zhu et al., 2014; Kato et al., 2015). The structural comparison between *Vibrio cholerae* DncV (VcDncV) and *E. coli* DncV (EcDncV) shows that their less conserved regions are flexible, and do not affect the overall structure, so the overall structure of DncV is fixed. DncVs from different bacterial species also produce bacterial-specific CDNs in a similar manner (Kato et al., 2015).

The core structure of DncV shows high similarity to that of cGAS with differences in a few loops (Zhu et al., 2014). The sequence of DncV and cGAS responsible for cGAMP generation is opposite (Schwartz et al., 2020). Most of the N-terminal extensions in DncV are part of the helical domain (HD), resulting in the helical domain forming a continuous hydrophobic core. The N-terminal extension of DncV plays an important role in the folding of the HD (Ming et al., 2014). The catalytic core of DncV is structurally conserved with that of the dsRNA sensor, OAS. Unlike cGAS and OAS, the structure of DncV remains in a self-activated state, while the activation of cGAS and OAS require the binding of dsDNA and dsRNA, respectively (Ming et al., 2014). The structure of the catalytic triplet of DncV in the free state is exactly the same as that of cGAS in the active state, indicating that the active site of DncV is formed well before the substrate binding or activator induction. This may explain why it no longer needs activation by inducers like dsDNA. Although DncV is present in an active conformation without binding to DNA, dsDNA can also bind to the back of the active site of DncV through complementary electrostatic interactions after comparing with the structure of dsDNA-activated cGAS. Therefore, the activity or function of DncV may be regulated by other potential factors. The crystal structure of human cGAS shows a positively charged fissure, which specifically binds to dsDNA and induces the rearrangement of cGAS structure along with its dimerization. As mentioned above, DncV does not need to bind dsRNA for its activation but DncV retains a long alkaline fissure and a conserved residue on the same side of the enzyme. The fissure and its function remain elusive to date (Davies et al., 2012; Kranzusch et al., 2014; Ming et al., 2014; Kato et al., 2015).

## 4. Synthesis and regulation of CDNs by DncV

Dinucleotide cyclase in *Vibrio* synthesizes three different CDNs, including c-di-AMP, c-di-GMP, and 3′3′-cGAMP *in vitro* but evidence suggests that DncV may not produce c-di-GMP *in vivo* (Kellenberger et al., 2013). Moreover, DncV produces 3′3′-cGAMP but not 2′,3′-cGAMP. The stimulatory activity of 3′3′-cGAMP weakens completely in cells expressing mutant STING (R231A), which can only recognize the ring dinucleotide with 2′,5′ bonds (Ablasser et al., 2013). This also indicates a certain difference in the function of cGAMP produced by the two. So how does DncV produce CDNs? Why is 3′3′-cGAMP produced preferentially? These questions need to be addressed further.

Dinucleotide cyclase in *Vibrio* contains receptor and donor nucleotide-binding pockets, which are recognized and bound by nucleotides and a substrate, GTP or ATP. The donor pocket similarly recognizes GTP and ATP, with a higher affinity for the former, thus supporting GTP as a donor nucleotide. For the receptor pocket, GTP and ATP are recognized differentially. The 3′-OH group of ATP is closer to the 3′-OH group of GTP (3.6 vs. 4.6 Å) with a better geometric configuration. Therefore, they tend to utilize ATP as a receptor nucleotide. DncV preferentially synthesizes 3′3′-cGAMP in the presence of both GTP and ATP because the binding affinity and reaction direction for the two substrates are different. Both GTP and ATP can be recognized by either pocket. Thus, the enzyme also cyclizes two ATPs or two GTPs to form c-di-AMP or c-di-GMP, respectively (Ming et al., 2014). The 3′-hydroxyl (OH) group of the receptor is closer to the α-phosphate group of the donor compared to the 2′-OH group, and thus, DncV catalyzes the formation of a 3′5′ phosphodiester bond in the first step of the reaction, rather than the 2′,5′ bond formed initially for cGAS.

The synthesis of cyclic dinucleotides by DncV can be controlled by folate-like molecules, 5-methyltetrahydro-folic acid (5MTHF), and 5-methyltetrahydrofolate diglutamate (5MTHFGLU2; Zhu et al., 2014). Folic acid binds to DncV in a similar pocket as that of dsDNA binding to cGAS. Receptor nucleotide orientation mainly determines the specificity of the unique linkage between DncV and cGAS. The combination of 5MTHF or 5MTHFGLU2 with DncV can stabilize the conformation of 110–119 residues and prevent the conformation conversion between different states, thus achieving the goal of inhibiting DncV enzymatic activity. Upon *Vibrio cholerae* infection, the decrease in folic acid uptake usually leads to prolonged diarrhea, and this phenomenon may be related to the regulation of DncV by folic acid. Together, these studies suggest an evolutionary trajectory, whereby metazoan cells adopted bacterial cyclase and developed a cytoplasmic DNA sensor tailored to their needs to defend against pathogens in the cell. This may also be because they are in the same or similar living environment, and the same or similar living pressure resulted in an enzymatic evolutionary convergence (Krasteva and Sondermann, 2017).

## 5. Role of DncV in antiphage immune system

Cyclic GMP-AMP synthesized by DncV is a part of the bacterial anti-phagocytic defense system against phage infection. Upon phage infection, the bacterial defense system is activated by four gene operons. DncV synthesizes and releases cGAMP, which in turn activates CapV phospholipase, leading to the degradation of the bacterial cell membrane (Figure 1; Severin et al., 2018; Cohen et al., 2019). Bacteria die before phage replication is complete. Owing to this suicidal death, the bacteria prevent phage proliferation, thereby protecting the entire flora. The defense system consists of four operons encoding DncV, CapV, and two additional genes encoding the protein domains, including E1, E2, and JAB. These domains are now known to be associated with the eukaryotic ubiquitin system akin to a deubiquitinase that removes ubiquitin from the target proteins. The activity of these two genes is necessary for defense against some phages but not all of them. This phage defense system is conducted by DncV and its homolog-related defense weapon, the CBASS. More than 10% of the bacterial genome contains this system and its variants; variants with effectors other than phospholipases also protect against phage infection (Severin et al., 2018; Cohen et al., 2019). Some phages have evolved mechanisms to circumvent CBASS by specifically degrading CDN signals that activate the host immunity (Duncan-Lowey and Kranzusch, 2022). Since CDNs produced by DncV and its homologs play an important role in bacteriophage defense, the discovery of homologs for other components of the eukaryotic immune system by evaluating the immune and anti-immune behaviors in prokaryotes is plausible.

## 6. Concluding remarks

As the important regulatory molecules of innate immunity, CDNs have been a research hotspot. Bartsch summarized the various biological and chemical synthesis pathways of CDNs such as 2′3′-cGAMP and 3′3’-cGAMP in detail (Bartsch et al., 2022). The yield of natural CDN from chemical synthesis is low and the synthesis route is complex. At present, gram-grade c-di-AMP can be prepared in the laboratory using immobilized *Vibrio cholerae* dinucleotide cyclase DncV, and can be used on a large industrial scale in the future with expected total yields of nearly 80%, thus facilitating the enzymatic synthesis of CDNs in large quantities (Sun et al., 2021; Bartsch et al., 2022). To obtain a variety of CDNs, different DncV homologs can be utilized for different purposes (Novotna et al., 2021). Therefore, the discovery of more DncV analogs and addressing the limitation of the stability of the enzyme synthesis process are warranted.

Compared to cGAS and OAS, DncV may be a primitive dinucleotide signaling synthase. Metazoans may have adopted a bacterial cyclase and have evolved immune systems to distinguish the recognition of dsDNA from dsRNA. In 2021, cGAS-like receptors (cGLRs) were found in *Drosophila*, which can sense dsRNA and generate the second messenger, 3′2′-cGAMP [cG(3′-5′)pA(2′-5′)P], to activate STING-dependent antiviral immune responses (Chen and Cao, 2021; Holleufer et al., 2021; Slavik et al., 2021). Interestingly, genes capable of synthesizing 3′2′-cGAMP have also been found in CBASS (Fatma et al., 2021), and thus, both bacteria and metazoans may have retained similar immune systems in the course of evolution. Moreover, cGAS and DncV have several structural and functional similarities. DncV and cGAS seem to constitute a new evolutionarily conserved group. The purpose of evolution is to detect the stimulation and/or changes in cells and their surroundings and generate corresponding responses. The appearance of DncV and cGAS in the two kingdoms of life could also be due to similar survival pressures or similar survival environments leading to the convergence of the two enzymes in the course of evolution. STING is present in bacteria and can recognize c-di-GMP (Morehouse et al., 2020). We compared the structural and functional differences between DncV and cGAS (Table 1). The evolution of bacterial immune systems for metazoan immunity is largely unclear and worthy of further investigation. Although DncV retains the basic fissure, the function of the fissure is unknown and is a potential research direction. Since DncV can affect several pathogenic bacteria, whether new antibacterial drugs can be designed according to this characteristic and is a new treatment direction against drug-resistant pathogenic bacteria, remain to be clarified. With the increase in knowledge of complex and diverse immune or defense systems of bacteria and the complex interactions between bacteria and host, understanding of these immune systems as the mirror image of the immune system of metazoans and the evolution of immune systems is facilitated and is further expected to pave the way in the study of the immune systems of metazoans.

## Author contributions

ZL wrote the manuscript. YF, XZ, and HD collected the literatures. ZL and QC revised the manuscript. All authors contributed to the article and approved the submitted version.

## Funding

This study was supported in part by the Natural Science Foundation of Fujian Province, China (Grant No. 2021J01206).

## Conflict of interest

The authors declare that the research was conducted in the absence of any commercial or financial relationships that could be construed as a potential conflict of interest.

## Publisher’s note

All claims expressed in this article are solely those of the authors and do not necessarily represent those of their affiliated organizations, or those of the publisher, the editors and the reviewers. Any product that may be evaluated in this article, or claim that may be made by its manufacturer, is not guaranteed or endorsed by the publisher.
